# Protein C Levels in Human Immunodeficiency Virus-Infected Women with and Without Pre-Eclampsia in South Africa

**DOI:** 10.3390/biomedicines14040866

**Published:** 2026-04-10

**Authors:** Wendy N. Phoswa, Lawrence Chauke, Kabelo Mokgalaboni, Gaynor Balie, Sidney Hanser, Olive P. Khaliq

**Affiliations:** 1 Department of Life and Consumer Science, College of Agriculture and Environmental Sciences, University of South Africa, Florida Campus, Roodepoort 1710, South Africa; mokgak@unisa.ac.za; 2 Department of Obstetrics and Gynaecology, School of Clinical Medicine, Faculty of Health Sciences, University of the Witwatersrand, Johannesburg 2193, South Africa; lawrence.chauke@wits.ac.za (L.C.);; 3 Department of Physiology, School of Medicine, Sefako Makgatho Health Science University, Ga-Rankuwa, Pretoria 0208, South Africa; sidney.hanser@smu.ac.za; 4 Department of Paediatrics and Child Health, School of Clinical Medicine, Faculty of Health Sciences, University of the Free State, Bloemfontein 9300, South Africa; khaliqop@ufs.ac.za

**Keywords:** anti-coagulants, HIV infections, pre-eclampsia, protein C

## Abstract

**Background:** Pre-eclampsia (PE) is a significant cause of maternal and perinatal morbidity and mortality globally and is characterized by impaired endothelial function and disturbances in coagulation pathways. The effects of Human Immunodeficiency Virus (HIV) on the immune and coagulation systems have been investigated during pregnancy, but there are few reports on anticoagulant factors in pregnant women who are infected with HIV and develop PE. This investigation compares plasma protein C levels in pregnant women with pre-eclampsia and those without pre-eclampsia, and compares the results based on their HIV status. **Methods:** A hospital-based cross-sectional study design was used for the current research, which was carried out at Charlotte Maxeke Johannesburg Academic Hospital, South Africa. A total of 83 pregnant women participated in the study and were categorized into one of four groups: normotensive HIV-negative (*n* = 36); normotensive HIV-positive (*n* = 18); pre-eclamptic HIV-negative (*n* = 21); and pre-eclamptic HIV-positive (*n* = 8). Data collected included demographic information and clinical characteristics that were abstracted from maternity records. Plasma protein C concentrations were determined by ELISA (enzyme-linked immunosorbent assay). Nonparametric statistical methods were used to compare the mean values of plasma protein C between each of the four groups, and significance was set at *p* < 0.05. Subgroup analyses, particularly for the pre-eclamptic HIV-positive group (*n* = 8), were considered exploratory due to small sample sizes. **Results:** As would be anticipated, both systolic and diastolic blood pressure values were significantly elevated in the pre-eclamptic group when compared to the normotensive control subjects (*p* < 0.0001). There were no statistically significant differences in plasma protein C concentration between the normotensive and pre-eclamptic groups, nor between the HIV-negative and HIV-positive groups. Similarly, there were no significant differences in plasma protein C concentration when comparing all four study groups (Kruskal–Wallis test *p* = 0.2295). **Conclusions:** Plasma protein C concentrations did not vary significantly according to the presence of pre-eclampsia or HIV status in this cohort. These findings suggest that protein C concentrations were not measurably altered between groups within this study population. However, due to the small sample size in key subgroups, these findings should be considered preliminary and interpreted with caution. Larger, adequately powered studies are required to further investigate potential associations between HIV infection, pre-eclampsia, and anticoagulant pathways during pregnancy.

## 1. Introduction

Pre-eclampsia (PE) is a pregnancy-specific hypertensive disorder occurring in approximately 5–7% of all pregnancies, and is responsible for significant maternal and perinatal morbidity and mortality [1]. The condition is usually defined by new onset hypertension at or beyond 20 weeks of gestation, often in conjunction with proteinuria or evidence of end-organ damage [2]. The pathogenesis of PE is complex and involves many different abnormalities of placentation, systemic inflammation, endothelial function and coagulation/vascular pathways [3,4,5,6,7,8]. These pathological processes may lead to a variety of maternal adverse outcomes, such as renal/hepatic/neurological dysfunction, and fetal adverse outcomes such as fetal growth restriction, premature delivery and stillbirth [9,10,11,12,13,14,15,16,17,18,19,20,21].

In sub-Saharan Africa, the impact of PE is compounded by the very high prevalence of Human Immunodeficiency Virus (HIV) infection amongst women of childbearing age [22,23,24]. Approximately 1.3 million women with HIV become pregnant annually according to the World Health Organisation (WHO) [25]. Highly active antiretroviral therapy (HAART) has greatly improved maternal survival rates and dramatically reduced mother-to-child transmission of HIV [25,26]. HAART may affect the regulation of the immune response and inflammatory pathways during pregnancy [27,28,29]. There is some conflicting evidence for an association between HIV infection/HAART and hypertensive disorders of pregnancy (HDPs), including PE [30,31,32,33].

Pregnancy is characterized by changes in the haemostatic system that favour a pro-coagulant state to reduce excessive bleeding at delivery [34]. This pro-coagulant state is counteracted by natural anticoagulant mechanisms that regulate thrombin generation and ensure vascular homeostasis. Abnormalities of this delicate balance may result in various pregnancy complications, including thrombosis, placental insufficiency and fetal growth restriction (FGR) [34]. Endothelial injury and disturbances in coagulation/fibrinolysis pathways have been shown to be involved in the development of pre-eclampsia and may result in a pro-thrombotic state [35,36,37].

Protein C is a Vitamin-K-dependent anticoagulant synthesized predominantly in the liver that plays a critical role in regulating coagulation through the inhibition of clotting factor Va and Factor VIIIa [38,39]. Therefore, protein C limits excessive thrombin formation and maintains haemostatic equilibrium. Altered protein C concentrations/activity have been reported in a number of pregnancy-related complications including pre-eclampsia; however, findings have been contradictory [40,41]. Decreased protein C levels have been reported in women with pre-eclampsia, indicating an impairment of regulatory anticoagulant mechanisms [40]; conversely, no significant difference in protein C levels between pre-eclamptic and normotensive pregnant women has been reported [41]. These contradictory findings suggest that the role of protein C in the pathogenesis of PE requires further clarification.

In addition to endothelial dysfunction caused by PE, HIV infection has also been demonstrated to cause chronic inflammation and alter coagulation pathways that could influence haemostatic regulation [29,42]. The effects of HIV infection on haemostasis could potentially interact with the pro-coagulant environment of pregnancy and influence anticoagulant profiles. Although there is potential for interaction between HIV infection and PE on coagulation pathways, relatively little research has investigated coagulation markers in pregnant women infected with HIV, especially in relation to PE [35,36,37,43].

Considering the very high prevalence of HIV infection amongst pregnant women in South Africa and the potential effects of both HIV infection and PE on coagulation pathways, further investigation of anticoagulant factors in this group is indicated. Limited data currently exist regarding protein C levels in HIV-infected women with PE.

This study therefore aimed to investigate and compare plasma protein C levels in pre-eclamptic women with and without HIV infection and in normotensive pregnant women attending a tertiary hospital in South Africa.

## 2. Materials and Methods

### 2.1. Study Population and Design

This is a hospital-based cross-sectional analytical study conducted at Charlotte Maxeke Johannesburg Academic Hospital (CMJAH). Institutional ethical approval was obtained for the study (University of South Africa—College of Agriculture & Environmental Sciences Health Research Ethics Committee (2022/CAES_HREC/005) and University of the Witwatersrand Human Research Ethics Committee (Medical) (M221163), South Africa). Additionally, regulatory permission was secured from CMJAH.

#### 2.1.1. Inclusion Criteria

Following the acquisition of written consent, women were consecutively recruited through convenience sampling according to pregnancy type (normotensive or pre-eclamptic) and HIV status at the time of presentation. A total of 29 pre-eclamptic and 54 normotensive women (aged 18 to 38 years) participated in the study. PE was defined as a new onset of blood pressure of ≥140/90 mmHg measured on two separate occasions four hours apart, with or without proteinuria. Normotensive participants were characterized by a blood pressure of ≤120/80 mmHg [44,45]. Demographic information for all participants was collected from their maternity case records. HIV testing was conducted after counselling using a rapid point-of-care test kit, following standard care procedures in South Africa. Maternal weight was classified into three categories: normal weight (BMI: 18–25 kg/m^2^), overweight (BMI: 25–30 kg/m^2^), and obese (BMI: >30 kg/m^2^). Furthermore, all HIV-positive participants were receiving HAART, which included tenofovir, emtricitabine, and efavirenz, in accordance with South African National HIV guidelines [27]. Highly active antiretroviral therapy (HAART) is the recommended treatment for HIV in both pregnant and non-pregnant women [30], as it significantly reduces the risk of mother-to-child transmission by lowering maternal viral load and providing pre- and post-exposure prophylaxis to the infant [26].

Participants were categorized not only by pregnancy type but also by HIV status, resulting in four groups: normotensive HIV-negative women (*n* = 36), normotensive HIV-positive women (*n* = 18), pre-eclamptic HIV-negative women (*n* = 21), and pre-eclamptic HIV-positive women (*n* = 8). All clinical and laboratory measurements were taken at a single time point, and participants were not monitored over time. However, clinical HIV-specific variables, including the duration of antiretroviral therapy, CD4 cell count, and plasma HIV viral load, were not consistently available from maternity case records at the time of recruitment and were thus excluded from the analysis. Consequently, adjustments for these HIV-related confounders could not be made.

#### 2.1.2. Exclusion Criteria

To ensure consistency in anthropometric measurements, women were excluded from the study if they had any pre-existing or concurrent chronic medical conditions, such as chronic hypertension, diabetes mellitus, renal disease, autoimmune disorders, cardiovascular disease, or known coagulation disorders. Additional exclusion criteria included multiple gestation pregnancies, current smoking, alcohol consumption, recreational drug use, and incomplete clinical records.

### 2.2. Quantification of Protein C

Maternal plasma samples, collected previously, were used to quantify protein C. Samples were obtained in Ethylenediaminetetraacetic acid (EDTA) tubes, centrifuged at 3000 revolutions per minute (rpm) for 10 min at 4 °C, and the isolated supernatant was stored at −80 °C until assayed.

The quantification of protein C using plasma samples was performed with the Human Coagulation Factor XIV/Protein C enzyme-linked immunosorbent assay (ELISA) Kit (catalogue number EH119RB) from Thermo Fisher Scientific (Waltham, MA, USA). Prior to analysis, samples were thawed completely on ice and mixed gently. For sample preparation, plasma was diluted 1000-fold using Assay Diluent C. The standards were prepared by reconstituting a lyophilized standard and creating a dilution series. The assay procedure included binding the antigen, adding a biotin conjugate, and then a Streptavidin–HRP solution, followed by the addition of tetramethylbenzidine (TMB) substrate and stop solution. For each step, there was incubation of 1 h, 45 min and 30 min, respectively. After incubation, the absorbance was read at 450 nm, and a standard curve was generated using curve-fitting software, followed by the calculation of protein C concentrations.

## 3. Statistical Analysis

Statistical analyses were performed using GraphPad Prism version 5.00 (GraphPad Software Inc., San Diego, CA, USA). Data distribution for each continuous variable was assessed prior to analysis. For variables with sample sizes greater than 30, the Kolmogorov–Smirnov test was used to evaluate normality, while the Shapiro–Wilk test was applied for variables with sample sizes ≤ 30. Variables were presented as median (Q1–Q3); mean ± SD is shown for descriptive purposes only.

Variables that followed a normal distribution were expressed as mean ± standard deviation (SD) and analyzed using parametric tests. Comparisons between two independent groups were performed using the unpaired Student’s *t*-test, and comparisons among more than two groups were conducted using one-way analysis of variance (ANOVA), followed by appropriate post hoc tests where applicable.

Variables that did not meet the assumptions of normality were expressed as median with interquartile range (IQR). For non-normally distributed data, comparisons between two groups were performed using the Mann–Whitney U test, while comparisons among more than two groups were carried out using the Kruskal–Wallis test followed by Dunn’s multiple-comparison post hoc test.

For each parameter, the choice of statistical test was determined based on the underlying data distribution. A *p*-value < 0.05 was considered statistically significant.

Categorical variables were summarized as frequencies and percentages. Comparisons between categorical variables were performed using the chi-squared (χ^2^) test when the expected frequency in each cell was ≥5. When the expected cell count was <5 in any category, Fisher’s exact test was applied. These approaches ensured the validity of statistical inference for categorical data.

### Power Analysis

An exploratory post hoc power analysis was conducted for this study, using G*Power (Version 3.1.9.7), to generate an approximate value of the statistical power of this study based on the current sample size. This post hoc power analysis indicated that the study had about 80% statistical power to detect medium effects in comparison among the study groups at an alpha level of 0.05; however, post hoc power calculations should be viewed with caution, since they are contingent upon both the observed sample size and effect size, and thus cannot replace an a priori sample size calculation.

Additionally, subgroup analyses in the current study were limited by very small sample sizes, specifically in the pre-eclampsia HIV+ group (*n* = 8). Thus, it is possible that the current study was underpowered to detect minor differences in plasma protein C levels among certain groups. These limitations should be taken into consideration when interpreting the results of this study and larger, better-balanced cohorts will be necessary to validate these observations in future studies.

## 4. Results

### 4.1. Clinical Characteristics of Participants

The clinical and demographic characteristics of the study population are summarized in Table 1. As expected, systolic and diastolic blood pressure were significantly higher in women with pre-eclampsia compared with normotensive controls (*p* < 0.0001).

No statistically significant differences were observed between the groups for maternal age, weight, height, body mass index (BMI), gestational age, or hemoglobin levels (all *p* > 0.05).

### 4.2. Concentration of Plasma Protein C

#### 4.2.1. Overall Comparison Across Study Groups

**Figure 1:**
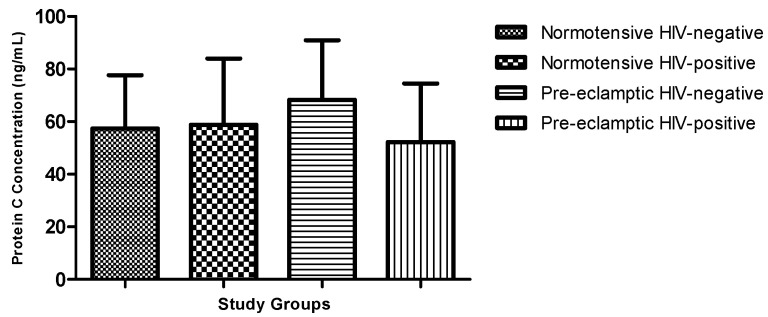
Plasma protein C across all study groups: normotensive HIV-negative; normotensive HIV-positive; pre-eclampsia HIV-negative; and pre-eclampsia HIV-positive.

Plasma protein C concentrations did not differ significantly across the four study groups (normotensive HIV-negative, normotensive HIV-positive, pre-eclamptic HIV-negative, and pre-eclamptic HIV-positive) (Kruskal–Wallis, *p* = 0.2295) (Figure 1).

#### 4.2.2. Comparison by Pregnancy Type

**Figure 2:**
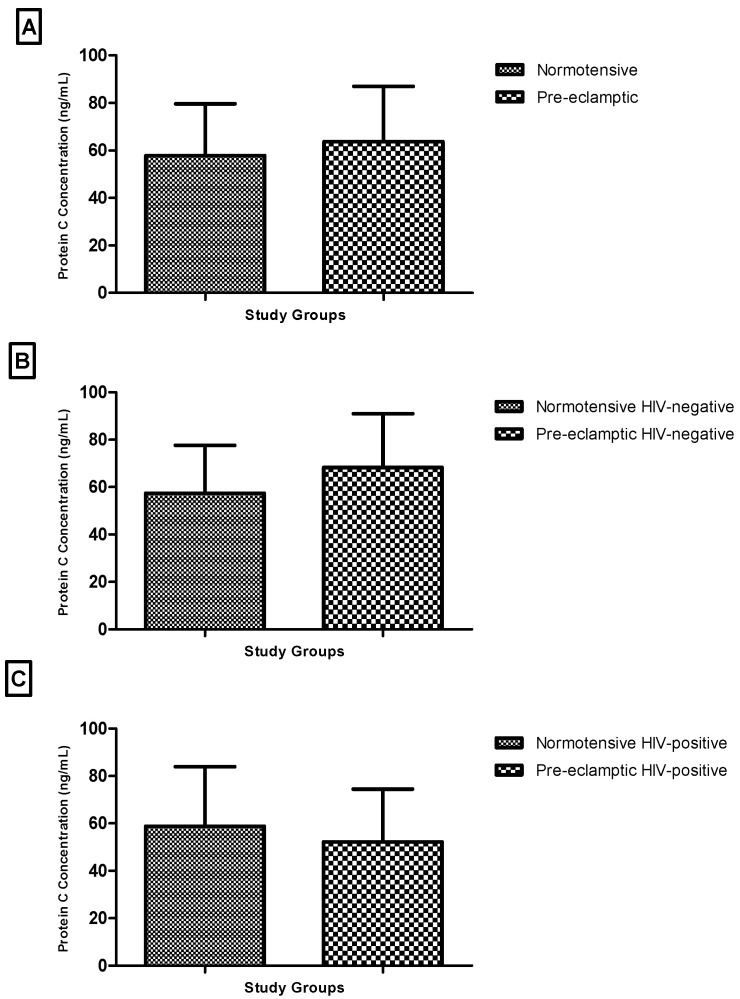
Plasma protein C based on pregnancy type: (**A**) normotensive vs. pre-eclamptic; (**B**) normotensive HIV-negative vs. pre-eclamptic HIV-negative; (**C**) normotensive HIV-positive vs. pre-eclamptic HIV-positive.

There was no statistically significant difference in plasma protein C levels between normotensive and pre-eclamptic women (Mann–Whitney U = 554.0, *p* = 0.7973) (Figure 2A).

Stratified analyses by HIV status also showed no significant differences. Among HIV-negative participants, protein C levels were similar between normotensive and pre-eclamptic women (*p* = 0.1259) (Figure 2B). Likewise, no significant difference was observed among HIV-positive participants (*p* = 0.3041) (Figure 2C).

It is important to mention that these subgroup analyses were based on relatively small sample sizes, particularly in the HIV-positive groups, and therefore may have limited statistical power to detect subtle or moderate differences. As such, the absence of statistically significant differences should be interpreted with caution and does not necessarily indicate equivalence between groups.

#### 4.2.3. Comparison by HIV Status

**Figure 3:**
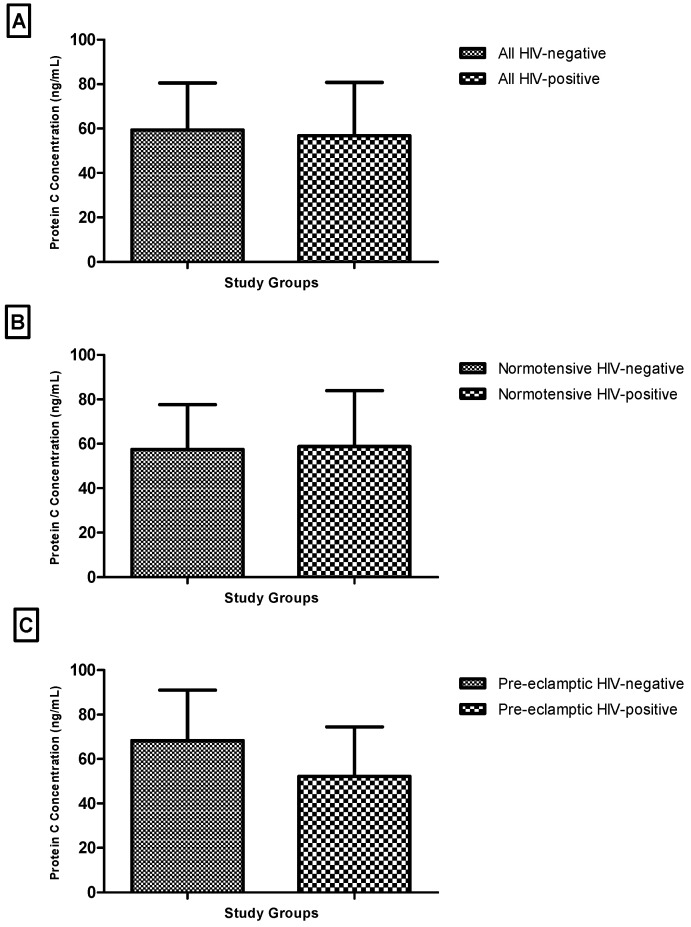
Plasma protein C based on HIV status: (**A**) all HIV-negative normotensive and pre-eclampsia vs. all HIV-positive normotensive and pre-eclampsia; (**B**) normotensive HIV-negative vs. normotensive HIV-positive; (**C**) pre-eclamptic HIV-negative vs. pre-eclampsia HIV-positive.

When grouped by HIV status, plasma protein C concentrations did not differ significantly between HIV-negative and HIV-positive participants overall (Mann–Whitney U = 482.0, *p* = 0.5158) (Figure 3A).

Similarly, no significant differences were observed within the normotensive subgroup (*p* = 0.9051) or the pre-eclamptic subgroup (*p* = 0.0703) (Figure 3B,C).

Again, it is important to note that these analyses, particularly within the pre-eclamptic subgroup, were constrained by small sample sizes, which may have reduced the ability to detect meaningful differences between HIV-positive and HIV-negative participants.

#### 4.2.4. Combined Comparisons of HIV Status and Pregnancy Type

**Figure 4:**
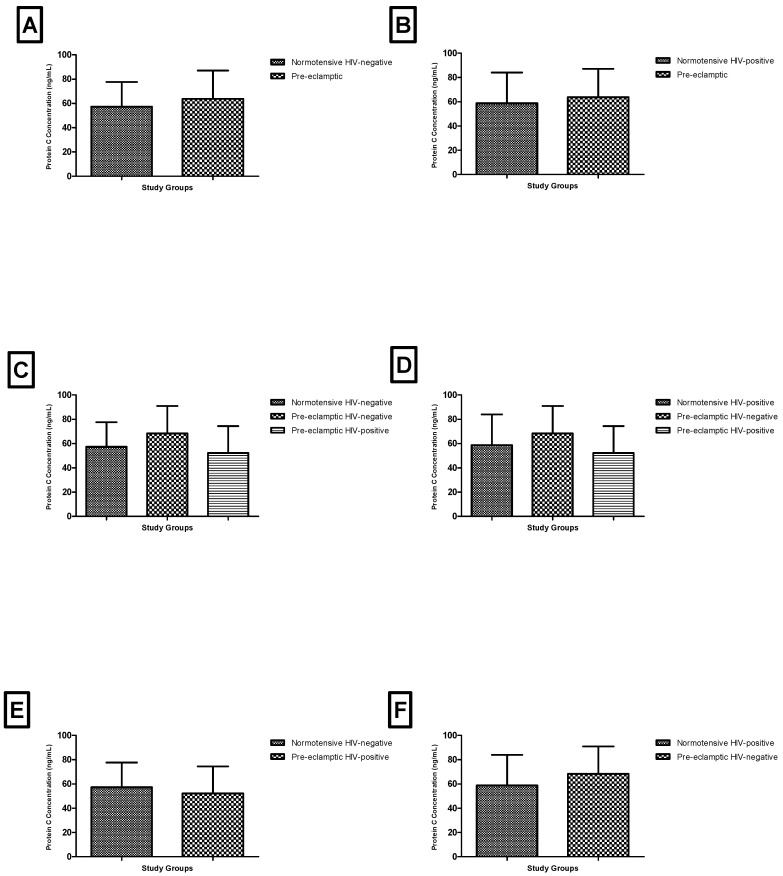
Plasma protein C based on HIV status and pregnancy type: (**A**) normotensive HIV-negative vs. all pre-eclampsia groups; (**B**) normotensive HIV-positive vs. all pre-eclampsia groups; (**C**) normotensive HIV-negative vs. pre-eclampsia HIV-negative vs. pre-eclampsia HIV-positive; (**D**) normotensive HIV-positive vs. pre-eclampsia HIV-negative vs. pre-eclampsia HIV-positive; (**E**) normotensive HIV-negative vs. pre-eclampsia HIV-positive; (**F**) normotensive HIV-positive vs. pre-eclampsia HIV-negative.

Additional comparisons across combinations of pregnancy type and HIV status did not reveal any statistically significant differences. Pairwise and multi-group comparisons, including Kruskal–Wallis analyses, were consistently non-significant (*p* > 0.05 across all comparisons) (Figure 4).

Given the very small sample sizes in several subgroups, particularly the pre-eclamptic HIV-positive group (*n* = 8), these analyses are likely underpowered and should be interpreted as exploratory. The lack of statistically significant findings in these comparisons may reflect limited statistical power rather than a true absence of biological differences.

#### 4.2.5. Summary of Findings

Overall, plasma protein C levels did not differ significantly according to pregnancy type, HIV status, or combinations of these variables within this study population.

Given the limited sample sizes in several subgroups, these findings should be interpreted cautiously, as the study may not have been sufficiently powered to detect small to moderate differences in protein C concentrations.

## 5. Discussion

This study evaluated plasma protein C concentrations in normotensive and pre-eclamptic pregnant women, stratified by HIV status. The primary finding was that protein C levels did not differ significantly according to pregnancy type, HIV status, or their combination, despite expected elevations in blood pressure in pre-eclamptic participants.

These findings are consistent with previous reports showing no significant differences in protein C levels between women with pre-eclampsia and normotensive pregnancies [43,46,47]. While pre-eclampsia is associated with endothelial dysfunction, systemic inflammation, and altered coagulation pathways [3,4,5,6,7,8,35,36,37,48,49,50], the present study did not detect measurable alterations in circulating protein C. Importantly, this study extends prior work by considering HIV infection as a potential modifier of coagulation, though no effect was observed. HIV infection has been reported to influence immune activation and coagulation [27,28,29,42], yet within this cohort, protein C concentrations remained similar across HIV-positive and HIV-negative groups.

The absence of statistically significant differences should be interpreted cautiously. The study may have been underpowered to detect subtle differences, particularly in small subgroups such as pre-eclamptic HIV-positive women (*n* = 8), increasing the risk of type II error. Physiological variation during pregnancy and broad gestational age ranges may also contribute to variability in protein C concentrations [34,51].

Additionally, key HIV-related clinical parameters—including CD4 count, viral load, and duration of antiretroviral therapy—were unavailable, limiting assessment of potential confounding or effect modification [27,28,29]. The cross-sectional design further restricts causal inference and prevents temporal assessment of changes in protein C. Measurement of protein C in isolation, without concurrent evaluation of other anticoagulants or endothelial markers, limits mechanistic insight into broader haemostatic changes.

Despite these limitations, the findings provide preliminary evidence that circulating protein C concentrations are not markedly altered by pre-eclampsia or HIV infection in this cohort. The results align with studies suggesting that protein C may not be a sensitive marker for pre-eclampsia-related coagulation changes [43,46,47]. Nonetheless, small subgroup sizes and missing clinical variables necessitate cautious interpretation, and the potential for subtle but clinically relevant differences cannot be excluded.

Future research should involve larger, well-powered cohorts, incorporate detailed HIV-related clinical data, and assess additional anticoagulant markers such as protein S and antithrombin, along with measures of endothelial function. Stratification by gestational age, pre-eclampsia severity, and antiretroviral therapy regimen will improve understanding of the interplay between HIV infection, hypertensive disorders of pregnancy, and haemostatic regulation. Such studies are essential to clarify whether anticoagulant pathways are differentially affected and to provide insight into potential mechanisms linking HIV infection and pre-eclampsia.

Within this study population, plasma protein C levels were not significantly altered by pre-eclampsia or HIV infection. The findings highlight the importance of adequately powered and well-characterized studies to explore haemostatic changes in complex clinical contexts.

## 6. Conclusions

In this study, plasma protein C levels were compared among normotensive and pre-eclamptic pregnant women with and without HIV infection. No statistically significant differences in protein C concentrations were observed between groups based on pregnancy type, HIV status, or their combination.

These findings suggest that, within this study population, circulating protein C levels were not significantly altered in association with pre-eclampsia or HIV infection. However, interpretation of these results should be made with caution due to the relatively small sample sizes in certain subgroups, particularly the HIV-positive pre-eclamptic group.

In addition, the cross-sectional design of the study and the absence of key HIV-related clinical variables, such as CD4 count, viral load, and duration of antiretroviral therapy, limited the ability to account for potential confounding factors. As a result, the present findings should be considered descriptive and do not support causal or mechanistic conclusions regarding the relationship between HIV infection, pre-eclampsia, and protein C regulation.

Further research with larger, well-characterized cohorts and comprehensive clinical data is needed to better understand potential interactions between HIV infection, hypertensive disorders of pregnancy, and haemostatic pathways.

### Limitations and Recommendations

A key limitation of this study is the relatively small sample size within certain subgroups, particularly the pre-eclamptic HIV-positive group (*n* = 8). The smaller sample size reduces the statistical power to detect subtle and potentially clinically relevant differences in protein C levels and increases the risk of type II error. As a result, the findings related to this subgroup should be interpreted with caution, as they may not be fully representative of the broader population of HIV-positive women with pre-eclampsia. Additionally, the small sample size limits the generalizability of the results and may partly explain the lack of statistically significant differences observed across groups. Future studies with larger, adequately powered cohorts are warranted to validate these findings and to better elucidate the interaction between HIV infection, pre-eclampsia, and anticoagulant regulation.

Furthermore, this study did not account for the duration of highly active antiretroviral therapy (HAART) or the specific drug regimens used, both of which may have differential effects on coagulation. An important limitation is also the lack of adjustment for key HIV-related confounding variables, including duration of antiretroviral therapy, CD4 count, and viral load. These parameters were not consistently documented in maternity records and were therefore unavailable for analysis. Given the known effects of immune status and chronic HIV-associated inflammation on hepatic function and coagulation pathways, their absence may have influenced the observed protein C levels, particularly among HIV-positive women with pre-eclampsia.

Another potential limitation of this study is the broad range of gestational ages among participants. Protein C levels are known to vary physiologically across trimesters, which may have affected the observed concentrations and contributed to variability in the results. Future research should aim for larger sample sizes and consider additional variables that could influence protein C levels, including population ethnicity and the severity of pre-eclampsia (mild, moderate, or severe), particularly among HIV-positive individuals. Moreover, future studies could explore other anticoagulants, such as protein S and antithrombin, while also examining the impact of various HAART regimens. Additionally, future studies can explore the relationship between protein C levels and obstetric outcomes. Such investigations would provide deeper insights into the role of this crucial anticoagulant during pregnancy and its involvement in the pathophysiology of pre-eclampsia, especially in the context of HIV infection.

The cross-sectional nature of the study further limits the ability to establish temporal or causal relationships between protein C levels, pre-eclampsia, and HIV status. Additionally, the use of convenience sampling at a single tertiary hospital may restrict the generalizability of the findings. Future studies should incorporate the aforementioned variables and apply multivariable statistical models to better delineate their independent and combined effects. Future studies should also consider stratifying participants by trimester or adjusting for gestational age in statistical analyses to more accurately isolate the effects of pre-eclampsia and HIV on protein C levels.

## Figures and Tables

**Table 1:** Summary of clinical and demographic features of the study population.

Variable	Normotensive (N) Median (IQR)	Pre-Eclampsia (PE) Median (IQR)	*p* Value
Maternal age (years)	34 (28–38)	34 (29–37)	0.9614
Maternal weight (kg)	73 (67.5–90)	85 (71–99)	0.2160
Maternal height (cm)	160 (155–163)	158 (155–160)	0.3838
BMI (kg/m^2^)	29.71 (27.15–36.73)	39.35 (25.56–42.17)	0.1051
SBP (mmHg)	110 (106.5–117.5)	154 (147–161)	*** <0.0001
DBP (mmHg)	67 (60.5–73)	101 (94–110.5)	*** <0.0001
Gestational age (weeks)	20 (15.5–29)	21 (15–28)	0.8905
Hemoglobin (g/dL)	10.95 (8.3–12.2)	11.05 (8.95–11.8)	0.6848

Data represented as median and interquartile range. *** *p* < 0.0001. N: normotensive; PE: pre-eclampsia; SBP: systolic blood pressure; DBP: diastolic blood pressure; BMI: body mass index; Hb: hemoglobin.

## Data Availability

The data that supports the findings of this study are available from the corresponding author upon request.
